# Impact of fully guided implant planning software training on the knowledge acquisition and satisfaction of dental undergraduate students

**DOI:** 10.1080/10872981.2023.2239453

**Published:** 2023-07-25

**Authors:** Shishir Ram Shetty, Colin Alexander Murray, Sausan Al Kawas, Sara Jaser, Natheer Al-Rawi, Wael Talaat, Sangeetha Narasimhan, Sunaina Shetty, Pooja Adtani, Shruthi Hegde

**Affiliations:** a College of Dental Medicine, University of Sharjah, Sharjah, United Arab Emirates; b College of Dentistry, Gulf Medical University, Ajman, United Arab Emirates; c Nitte (Deemed to Be University), Mangalore, India

**Keywords:** implant planning software, knowledge acquisition, satisfaction, dental students, education

## Abstract

**Background:**

A majority of dental school students do not undergo hands-on clinical training in implantology in the undergraduate curriculum. Training is usually restricted to pre-implant evaluation and post-implant prostheses. Virtual implant planning software (VIPS) provides an alternative opportunity for undergraduate students to experience implant planning much before gaining hands-on experience. However, not many studies have the contribution of VIPS to the knowledge acquisition of students. We conducted a preliminary study to evaluate the knowledge acquisition of the students when exposed to a hands-on session of VIPS. We also evaluated students’ satisfaction levels, when exposed to hands-on training in fully guided implant planning software.

**Methods:**

A two-part theory lecture on fully guided implant planning was delivered to 90, 5th (final)-year dental undergraduate students by the oral radiology faculty. The students were then randomly divided into three groups. Group A was exposed to didactic lectures only. Group B was shown a video for fully guided implant planning in addition to the didactic lecture. Group C was shown a video for fully guided implant planning in addition to a didactic lecture and then performed a hands-on session of virtual implant planning under faculty guidance. Students from all groups were given an MCQ-based test. After the completion of the test students from group A and B also received VIPS hands-on training. Students from all three groups answered and a feedback questionnaire regarding their satisfaction levels with VIPS.

**Results:**

The overall test score of students in Group C was higher than their colleagues in both Groups A and B and the differences were statistically significant (*p* = 0.01). More than 85% of the students were satisfied with the teaching approach.

**Conclusions:**

The utilization of VIPS in the training of dental undergraduate students improves their performance confirming better knowledge acquisition and content mastery.

## Introduction

With the advancement of information technology, virtual simulation has become one of the newer methods of teaching during practical sessions [1]. Virtual reality technology has made significant progress due to its advantages of immersion, interactivity, and independence [2]. It also offers a novel direction for integration and utilization in different disciplines [3]. The utilization of virtual reality in teaching curricula has been proven to be efficient in improving students’ skills [4]. In the medical field, the use of high simulation devices to complement conventional training approaches has long been the vision of medical educators [2]. The simulation aims to copy real clinical set-ups and to reflect real-life situations in which medical services are offered. Simulation is specifically essential in understanding complex spatial relations of anatomical structures and in assessing surgical methods. The combination of virtual reality technology and medicine will be the new direction in the training of medical students [5].

Traditionally, conventional radiographs have been utilized to view the complex internal structures of the human teeth and jaw [6]. The advent of cone beam tomography (CBCT) and the invention of interactive software to allow virtual planning to guide surgery accurately towards a specific target has greatly enhanced dental oral surgery [6]. VIPS provides prosthetically driven approaches resulting in better prosthesis design and esthetics-optimized occlusion [7]. The software also allows the operator to mark important anatomical landmarks such as the inferior alveolar canal on the CBCT scans [7].

There are two distinct categories of guided implant surgery, that is, static and dynamic. The static method or computer-guided surgery involves the use of tissue supported template [8]. This generates the virtual implant position right from computerized tomographic data. The data is then transformed to guide templates for utilization during surgery [8]. Conversely, dynamic, or navigation-guided surgery generates virtual implant positions directly from computerized tomographic data and employs motion-tracking methods to drive the implant process [8]. With further advancements in technology, different levels of evidence were presented that showed various degrees of precision [8].

An intraoral scan can capture the shape, form, and structures of the teeth and soft tissues. A combination of cone beam tomography and intraoral scan images by mutual superposition and the use of virtual implant planning presents a 3D depiction of hard and soft tissue [8]. Moreover, the novel planning software allows nurturing of a digital wax-up of the future prosthetic plan which can be modified whenever necessary.

Many dental colleges frequently adopt new teaching technologies like virtual implant planning software (VIPS) [9]. A position paper peer reviewed by experts in the field of undergraduate education in implant dentistry recommended that “universities should report comprehensively on their undergraduate implant training to allow comparison and reproduction in other environments [10]. Despite the large volume of work in this area, only limited research has been conducted in the field of dental education. At our university, implantology in the undergraduate dental curriculum is taught as a part of oral surgery, oral radiology, and prosthodontic courses and not as an individual course. Therefore, using the VIPS would help students understand both the theoretical concepts and its applications. We conducted a preliminary study to evaluate the impact of exposure to virtual implant planning video and hands-on training versus exposure to virtual implant planning video and didactic lectures on the knowledge acquisition of the learners. We also assessed student satisfaction after exposure to the virtual implant planning software.

## Materials and methods

**Figure 1.**
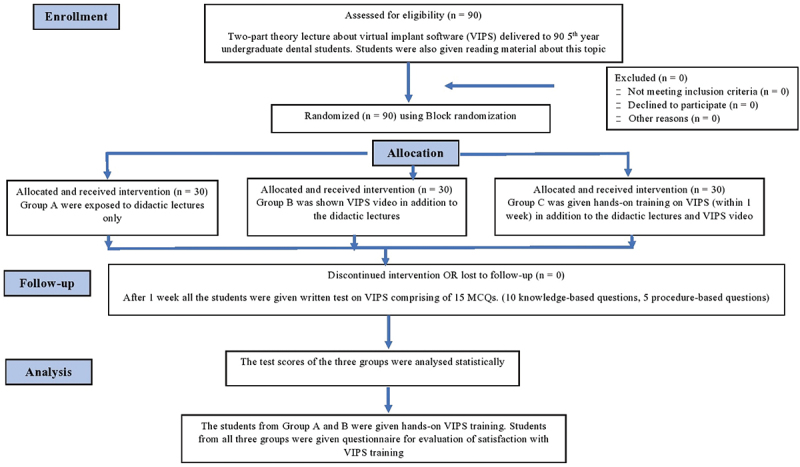
CONSORT flowchart of the methodology followed in the study.

The study was conducted on 90 final-year BDS students, after approval by the Research Ethics Committee. Written informed consent was obtained from all participants. A two-part theory lecture of one hour each on fully guided implant planning was delivered to final-year dental students by oral radiology faculty in the month September 2022. The first part of the lecture included the basics of implant planning. It had information on the importance of implant planning, types of implant planning, implant centric view of CBCT, and method of obtaining intraoral scans. In the second part of the lecture, step-by-step procedure of the implant planning was explained to the students using screenshots from the Planmeca software. The Powerpoint presentations were handed over to all the students. Later, they were randomly divided into 3 groups A, B and C. Each group consisted of 30 students. Block Randomization was used to create the allocation list for the three study groups. Randomization sequence was created using Excel 2011 (Microsoft, Redmond, WA, USA) with random block sizes of six and equal allocation ratio by an investigator with no involvement in the trial. The allocation sequence was concealed from the investigators enrolling and assessing participants (students) in sequentially numbered, opaque, sealed, and stapled envelopes. The envelopes were opened at the time of intervention. Group A was exposed to didactic 2-part lectures only. Group B was shown a video for fully guided implant planning in addition to the 2-part didactic lectures they attended within a week. Group C was shown a video for fully guided implant planning in addition to a didactic lecture. Group C additionally performed a hands-on session of virtual implant planning under faculty guidance within one week. (Figure 1). A priori power analysis was conducted using G*Power version 3.1.9.6 for sample size estimation, based on data from previous literature [1,11]. Considering an effect size of 0.35, α = 5% and power = 80%, the minimum sample size needed was 28 per group, which was rounded up to 30 participants.

**Figure 2.**
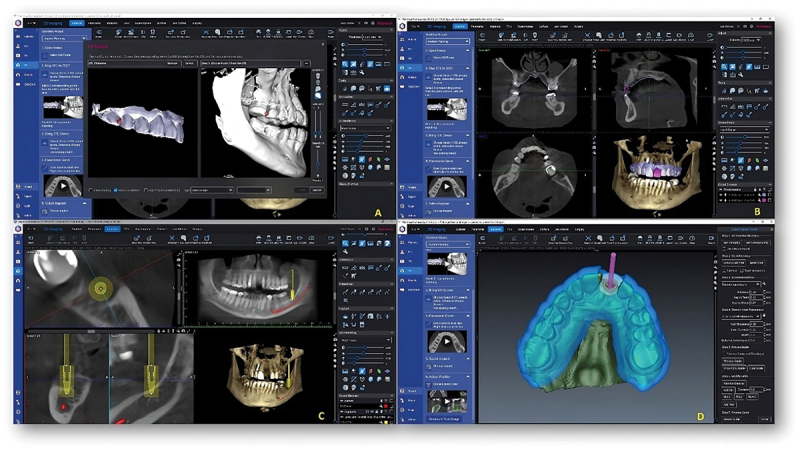
Showing the basic steps in virtual implant planning procedure. A-Mapping the intraoral scan with CBCT. B-Mapping of virtual crown with CBCT. C- position the implant using implant centric view. D- preparation of the virtual surgical guide.

The Romexis 6.2.1 version of virtual implant planning software (Planmeca Viso 7 CBCT unit, Finland) was used for the hands-on session. The hands-on session started with mapping of intraoral scan with CBCT. This was followed by mapping of virtual crown with CBCT scan. Then reformatted panoramic curve was made followed by implant selection and placement. This was followed by preparation of virtual surgical guide and comprehensive implant report (Figure 2).

A week after the didactic lecture, all the students were given an online test of 15 Multiple Choice Questions (MCQs) on the topic of fully guided implant planning using Blackboard Ultra (Blackboard Inc, Washington, USA). The MCQs were constructed by two faculties with 10 years of clinical and teaching experience in implant radiology. The MCQs were then revised and validated by another subject expert with similar experience in this field. Among the 15 MCQs, 10 of them were related to the basics (knowledge component, knows) of virtual implant planning. Five MCQs were focused on the sequence of events (procedural component, knows how) in the virtual implant planning (Appendix 1).

The discrimination index and difficulty level of the MCQ items were analyzed using blackboard analytics. According to blackboard analytics the scales of the discrimination values range from −1.0 to + 1.0. Values above 0.30 is good, whereas values between 0.10 and 0.30 are fair. The question is considered to be a poor discriminator if the values range from −1.0 to 0.10. Discrimination cannot be ascertained when a question has difficulty score of 100% or all students get same score on a particular question [12]. Difficulty index is assessed based on the number of students who answer the question correctly. The values can range from 0 to 100% [13]. The reliability of the questions was assessed using the Cronbach’s Alpha. Values above 0.9 was considered to have excellent. The values are interpreted as good if the values are greater than or 0.8 but less than or equal to 0.9. Values below 0.8 are further classified as acceptable, questionable and poor [14].

The student scores were compared between the three groups using statistical tests. Students from groups A and B were then given hands-on training in fully guided implant planning. Finally, students from all three groups (A, B and C) were asked to fill out a questionnaire regarding their satisfaction levels with fully guided implant planning. The questionnaire used for the study was adapted from the study by Lu et al [1]. The questions relate to students’ understanding of theoretical and practical knowledge, familiarity, convenience, and likeability of the fully guided implant planning software. Responses to questions range from Strongly agree or slightly agree or disagree [1]. Additionally, there was also a section for any other comments that students could fill out voluntarily.

### Statistical analysis

The data obtained from the tests were analyzed using the IBM SPSS statistics (Version 22, IBM Corp,Armonk. NY, USA). The overall score, scores in the knowledge component and scores in the procedural component among the three study groups were compared using the ANOVA and Tukey Post Hoc Test. *P* < 0.05 was considered statistically significant.

## Results

The overall test score of Group C was higher than that of Group A and group B. Similarly Group C had higher test scores in the knowledge and the procedure components of the MCQs, when compared to Group A and Group B. Overall comparison of the scores using ANOVA test showed a significant difference (*p* = 0.01) among the three groups (Table 1).

**Table 1.** Overall Comparison of the test scores between the study groups.

							ANOVA	
	Study Group	N	Mean	SD	Minimum	Maximum	F	p-value
Knowledge component (Knows) (out of 10)	A	30	7.27	1.36	5	10	24.44	0.01*
	B	30	7.30	1.56	4	10		
	C	30	9.27	0.74	8	10		
Procedure component (Knows how) (out of 5)	A	30	3.17	1.02	1	5	15.09	0.01*
	B	30	2.97	1.25	1	5		
	C	30	4.30	0.70	3	5		
Overall test score (out of 15)	A	30	10.57	1.68	8	14	31.04	0.01*
	B	30	10.33	2.26	6	15		
	C	30	13.57	1.22	11	15		

Note: *p < 0.05 Statistically Significant, *p* > 0.05 Non-Significant, NS.

Pairwise comparison using Tukey Post Hoc Test revealed that Group C students had significantly (*p* = 0.01) better scores than Group A and B (Table 2). This shows that students with hands-on experience of fully guided implant planning had better understanding of the concept compared to students who attended the lecture sessions and watched demonstration video. However, there was no significant difference between the test scores of the Group A and Group B (*p* = 0.87). This indicates that there was no significant knowledge acquisition in the students who watched demonstration videos in addition to lectures.

**Table 2.** Pairwise comparison of the test scores between the study groups.

						95% Confidence Interval	
	(I) Groups	(J) Groups	Mean Difference (I-J)	Std. Error	p-value	Lower Bound	Upper Bound
Knowledge component (Knows) (out of 10)	A	B	−0.03	0.33	0.99(NS)	−0.81	0.75
		C	−2.00	0.33	0.01*	−2.78	−1.22
	B	C	−1.97	0.33	0.01*	−2.75	−1.19
Procedure component (Knows how) (out of 5)	A	B	0.20	0.26	0.73(NS)	−0.42	0.82
		C	−1.13	0.26	0.01*	−1.76	−0.51
	B	C	−1.33	0.26	0.01*	−1.96	−0.71
Overall test score (out of 15)	A	B	0.23	0.46	0.87(NS)	−0.86	1.33
		C	−3.00	0.46	0.01*	−4.09	−1.91
	B	C	−3.23	0.46	0.01*	−4.33	−2.14

Note: Tukey Post Hoc Test.

*p < 0.05 Statistically Significant, *p* > 0.05 Non Significant, NS.

### MCQ analysis

Blackboard question analysis revealed that the majority (*n* = 9) of the questions had fair discrimination and medium difficulty (*n* = 10). The Chronbach’s alpha value for the MCQs was 0.866 indicating good reliability (Table 3).

**Table 3.** Question analysis of the MCQs used in the study.

Discrimination	Range	Number of questions
Good	>0.30	4
Fair	0.10 to 0.30	9
Poor	−1.0 to 0.10	2
Can’t calculate		0
Total questions		15
**Difficulty**	**Range**	**Number of questions**
Easy	>80%	5
Medium	30% to 80%	10
Hard	<30%	0
Total questions		15
**Reliability**	**Range**	**Cronbach’s Alpha**
**Good**	0.8 to 0.9	0.866

### Feedback from Questionnaires

**Figure 3.**
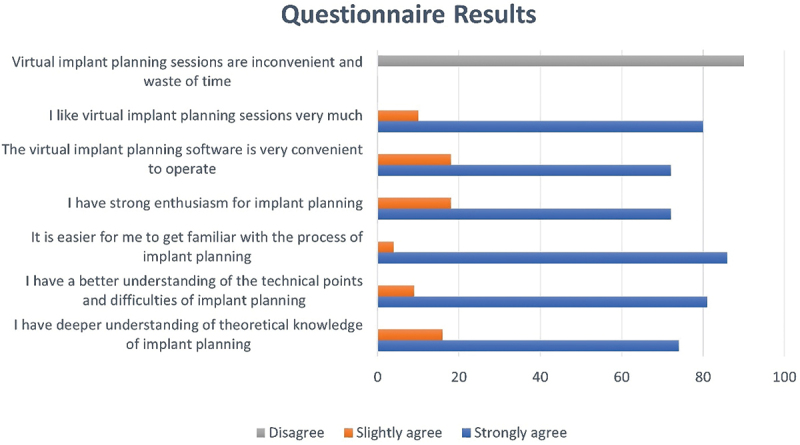
Graph showing results of the questionnaire regarding fully guided implant planning.

**Figure 4.**
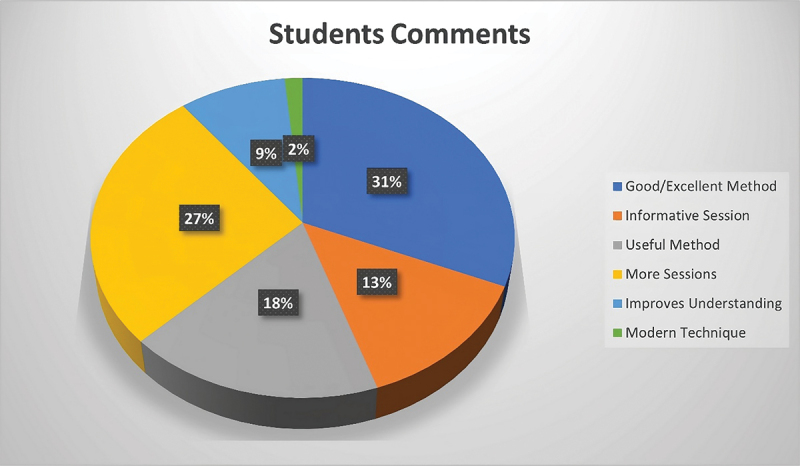
Pie chart showing students’ views on virtual implant planning.

Majority of the students (*n* = 85, 94.44%) strongly agreed that VIPS made it easier for them to familiarize with the process of implant planning. Similarly, 90% (*n* = 81) of the students strongly agreed that it was easier for them to get an understanding of the technical points and difficulties of implant planning with VIPS. The number of students strongly agreeing that they liked VIPS training sessions was 80 (88.88%). None of the students found the virtual implant planning inconvenient or a waste of time and therefore, was generally well accepted. The results are shown in Figure 3. A few of the students provided additional views on the use of virtual implant planning for teaching purposes. They felt that the sessions were good, useful, informative, and improved understanding. They also recognized that it was a modern technique and anticipated for more sessions. Students’ views are summarized in Figure 4.

## Discussion

In the present study VIPS training was given to final year BDS students (advanced beginners according Modified Dreyfus Model). The educational strategy was to assess the students ‘knows’ and ‘knows how’ components (Modified Millers model) of implant planning using VIPS (simulation) [15]. The virtual implant planning experimental environment was based on computer simulation and had two modes of operation. In learning mode, students could simulate the whole process of reception, evaluation, and treatment according to the video prompts on toolbar. In the practice mode, users performed hands-on training in fully guided implant planning. The practice mode permitted multiple repetitions which enhances content acquisition, retention, and mastery [16]. Instructors could use the VIPS to monitor students’ learning time, progress, and content mastery to advance teaching effectiveness [17].

The findings of this study demonstrated that hands-on VIPS training significantly improves students’ knowledge acquisition. Students belonging to group C (didactic lecture + video session + hands-on VIPS session) performed significantly better than the group A and B in both knowledge component (*p* = 0.01) and procedure component (*p* = 0.01). Similar results were obtained in the study by Lu et al [1]. In their study, the group of students exposed to virtual pulpotomy teaching platform had better (*P* < 0.05) theoretical scores than the group of students with no exposure [1].

It is also worth noting that students who were subjected to videos for fully guided implant planning in addition to a didactic lecture (Group B) scored relatively higher than their batchmates who were only subjected to didactic lectures only (group A). However, the difference was not statistically significant (*p* = 0.87) indicating that visual demonstrations have a limited role to play in the training of dental students. This comparison was not done in the study by Lu et al since it involved only two groups (video demonstration only and video demonstration + virtual simulation) [1].

Furthermore, previous research has also shown that virtual simulation plays a central role in medical training, assessment, and self-learning [16–22]. Therefore, virtual simulation improves not only learning performance but also teaching efficiency, confirming that the training method is effective and worth adopting [16–22].

The feedback from the questionnaires, revealed that 94.44% strongly agreed that VIPS made it easier for them to familiarize with the process of implant planning. In the study by Lu et al et above 90% of the students strongly agreed that the virtual simulation session made it easier for them to familiarize with the endodontics and pulpotomy [1]. No student found the virtual implant planning inconvenient or a waste of time which was similar to the findings in the study by Lu et al [1].

Students enrolled on the virtual implant planning platform enjoy immense educational benefits; they preview lessons before, during and after classes or at any time on the platform [1]. This promotes efficient utilization of teaching resources and resource sharing. Unlike conventional teaching methods, the virtual simulation teaching technique assists students to develop their own thinking, encourages self-learning and self-assessment, practical skill, improves the mastery of necessary content, and promotes exploration [1].

In comparison with conventional evaluation techniques, the virtual simulation teaching platform is more pragmatic and precise and measures training, and evaluation and consistently make improvements according to the instructor feedback [16,17]. Recently, virtual simulation has been utilized in dental education as an adjunctive to the conventional skill training curriculum to train dentists before interacting with real patients [23,24]. Dental education differs greatly from other forms of medical education since it is a blend of theory, practical and clinical practice. The challenge in training dentists arises from the fact that at times even theoretical knowledge acquisition needs spatial imagination of structures and procedures [25]. Virtual simulation offers a solution to this problem, thereby improving theoretical understanding in addition to improving preclinical skills [25]. Furthermore, preclinical and clinical training is of utmost necessity for nurturing motor skills to prepare dental students to engage in dental professional practice. Most of the dental education competency skills required are difficult to acquire and mandate repeated training and a long practice [26].

Medical and dental students find it relatively easy to familiarize themselves with novel technologies without having to undergo rigorous training unlike general populations [27,28]. Dental students thus will be able to overcome the inevitable drawbacks of the implementation of virtual implant planning in both undergraduate and postgraduate curricula [29,30]. Moreover, various undergraduate and post-graduate programs incorporate virtual technologies to aid in teaching and learning dental surgery, because there are no clinical consequences associated with virtual technologies [29,30]. Furthermore, virtual technologies provide platforms for multiple repetitions which is crucial for learning and practice of novel concepts [31].

### Limitations

One of the major limitations of the study is that long-term retention of knowledge has not been evaluated. Another limitation of the present study is that the number of MCQs are less. Lastly, the study was restricted to the student cohort of a single university only. Study involving students from multiple dental schools can be considered in the future.

### Future recommendations

Conduct follow-up studies should be performed to evaluate the long-term knowledge retention of the learners/students. 2) Introduce virtual implant planning software videos and hands-on training synergistically while teaching the theoretical concepts of implantology as a part of oral surgery, oral radiology, and prosthodontics. 3) Introduce integrated sessions with all clinical discipline’s involved in teaching implantology.

## Conclusions

Applying information technology to the practical aspects of training, creating a flexible and networked virtual simulation teaching platform, and establishing a training model can effectively compensate for the deficiencies of conventional practical teaching. A hybrid system of teaching dental students that incorporates didactic lectures, video demonstrations and hands-on sessions of virtual implant planning has the greatest impact on knowledge acquisition, retention, and mastery of content. The utilization of virtual implant planning in the training of dental undergraduate students improved their performance scores. We suggest that virtual implant planning should be introduced in the curriculum of undergraduate dental surgery programs.

## Supplementary Material

Supplemental Material

## Data Availability

The raw data is available at figshare; 10.6084/m9.figshare.22059254

## Appendix 1. MCQs used for the evaluation of knowledge regarding fully guided virtual implant planning

**Table:**

Sl no	MCQ stem	Possible answers/choices
1	What is the format of the intraoral scan used in virtual implant planning?	STL JPEG PNG TIFF
2	What is the primary function of the implant sleeve in implantology procedure?	To stabilize the implant To guide/position the surgical drill To guide/position the surgical drill and subsequent implant To dissipate heat produced during implant drill
3	Which of the below mentioned options is one of the advantages of fully guided implant procedure?	Prosthesis driven implant can be achieved Implant driven prosthesis can be achieved Implants can be loaded immediately Requires less implant drilling time
4	What is the minimum distance between tooth and dental implant?	1 mm 1.5-2 mm 3-4 mm 4-5 mm
5	What is the minimum distance between two dental implants?	1 mm 1.5-2 mm 3-4 mm 4-5 mm
6	In case of the infringement of safe zone around the virtual implants … … .	The softwares alerts the operator The software repositions the virtual implant The software does not show any alert The software automatically reduces the dimensions of the implant
7	What is an implant centric view?	Oblique cross-sectional view that always pass through the long axis of the implant Oblique cross-sectional view that always pass through the short axis of the implant Oblique cross-sectional view that always pass through the long axis of the edentulous area Oblique cross-sectional view that always pass through the short axis of the edentulous area
8	What are minimum number of points require for mapping STL files to CBCT landmarks in virtual implant planning software?	Two Three Four Five
9	The report generated by the virtual implant software includes … … … …	Patent details and operator’s findings only Patent details, operator’s findings and implant centric view only Patent details, operator’s findings, implant centric Implant centric view and implant dimension Patent details, operator’s findings, implant centric Implant centric view, implant dimensions and drill length estimation
10	In which format is the virtual surgical guide exported for 3D printing?	DICOM file STL file JPEG file TIFF file
11	Which of the component needs to be hidden before drawing the surgical guide area during virtual implant planning?	STL file of the crown STL file of the intraoral scan STL files of the crown and intraoral scan DICOM file of the CBCT scan
12	Identify the correct sequence of events in implant selection using the below mentioned options Select appropriate implant based on length and diameter Open virtual implant library Confirm selection by checking the 3 images of the implant and sleeve Select appropriate sleeve	2,1,4,3 2,1,3,4 1,2,3,4 4,2,3,1
13	Identify the correct sequence of steps in generating a reformatted panoramic curve Mark the reference points on the axial CBCT section for generating the curve Determine an adequate axial CBCT section to view the arch. Confirm the reformatted panoramic view on the other window	2,1,3 1,2,3 3,2,1 2,3,1
14	Identify the correct sequence of events in preparing virtual surgical guide from the below mentioned options (1) Select guide thickness (2) Add support bar and label guide (3) Draw guide area on virtual cast	3,1,2 3,2,1 1,2,3 2,1,3
15	Identify the correct sequence of events in implant planning from the below mentioned options (1) Select implant (2) Map STL of intraoral scan with CBCT (3) Map STL of virtual crown with CBCT (4) Draw panoramic curve (5) Export STL of surgical stent (6) Check implant position (7) Draw surgical guide	1,2,3,4,5,6,7 2,3,4,1,6,7,5 4,3,6,7,1,2,5 2,6,7,4,3,5,1
	Key answers- 1)-a, 2)-c, 3)-a, 4)-b, 5)-c, 6)-a, 7)-a, 8)-b 9)-d, 10)-b, 11)-a, 12)-b, 13)-a, 14)-a, 15)-b	
