# Supplementary material for: Impact of fully guided implant planning software training on the knowledge acquisition and satisfaction of dental undergraduate students
Source: Med Educ Online. 2023 Jul 25;28(1):2239453. doi: 10.1080/10872981.2023.2239453 (PMC10392243; doi:10.1080/10872981.2023.2239453)
Supplement: Supplemental Material [file ZMEO_A_2239453_SM9654.docx]

Appendix 1. MCQs used for the evaluation of knowledge regarding fully guided virtual implant planning

| Sl no | MCQ stem | Possible answers/choices |
| --- | --- | --- |
| 1 | **What is the format of the intraoral scan used in virtual implant planning?** | 1. STL 2. JPEG 3. PNG 4. TIFF |
| 2 | **What is the primary function of the implant sleeve in implantology procedure?** | 1. To stabilize the implant 2. To guide/position the surgical drill 3. To guide/position the surgical drill and subsequent implant 4. To dissipate heat produced during implant drill |
| 3 | **Which of the below mentioned options is one of the advantages of fully guided implant procedure?** | 1. Prosthesis driven implant can be achieved 2. Implant driven prosthesis can be achieved 3. Implants can be loaded immediately 4. Requires less implant drilling time |
| 4 | **What is the minimum distance between tooth and dental implant?** | 1. 1mm 2. 1.5-2mm 3. 3-4mm 4. 4-5mm |
| 5 | **What is the minimum distance between two dental implants?** | 1. 1mm 2. 1.5-2mm 3. 3-4mm 4. 4-5mm |
| 6 | **In case of the infringement of safe zone around the virtual implants…….** | 1. The softwares alerts the operator 2. The software repositions the virtual implant 3. The software does not show any alert 4. The software automatically reduces the dimensions of the implant |
| 7 | **What is an implant centric view?** | 1. Oblique cross-sectional view that always pass through the long axis of the implant 2. Oblique cross-sectional view that always pass through the short axis of the implant 3. Oblique cross-sectional view that always pass through the long axis of the edentulous area 4. Oblique cross-sectional view that always pass through the short axis of the edentulous area |
| 8 | **What are minimum number of points require for mapping STL files to CBCT landmarks in virtual implant planning software?** | 1. Two 2. Three 3. Four 4. Five |
| 9 | **The report generated by the virtual implant software includes…………** | 1. Patent details and operator’s findings only 2. Patent details, operator’s findings and implant centric view only 3. Patent details, operator’s findings, implant centric Implant centric view and implant dimension 4. Patent details, operator’s findings, implant centric Implant centric view, implant dimensions and drill length estimation |
| 10 | **In which format is the virtual surgical guide exported for 3D printing?** | 1. DICOM file 2. STL file 3. JPEG file 4. TIFF file |
| 11 | **Which of the component needs to be hidden before drawing the surgical guide area during virtual implant planning?** | 1. STL file of the crown 2. STL file of the intraoral scan 3. STL files of the crown and intraoral scan 4. DICOM file of the CBCT scan |
| 12 | **Identify the correct sequence of events in implant selection using the below mentioned options**   1. **Select appropriate implant based on length and diameter** 2. **Open virtual implant library** 3. **Confirm selection by checking the 3 images of the implant and sleeve** 4. **Select appropriate sleeve** | 1. 2,1,4,3 2. 2,1,3,4 3. 1,2,3,4 4. 4,2,3,1 |
| 13 | **Identify the correct sequence of steps in generating a reformatted panoramic curve**   1. **Mark the reference points on the axial CBCT section for generating the curve** 2. **Determine an adequate axial CBCT section to view the arch.** 3. **Confirm the reformatted panoramic view on the other window** | 1. 2,1,3 2. 1,2,3 3. 3,2,1 4. 2,3,1 |
| 14 | **Identify the correct sequence of events in preparing virtual surgical guide from the below mentioned options**  **1. Select guide thickness**  **2. Add support bar and label guide**  **3. Draw guide area on virtual cast** | 1. 3,1,2 2. 3,2,1 3. 1,2,3 4. 2,1,3 |
| 15 | **Identify the correct sequence of events in implant planning from the below mentioned options**  **1. Select implant**  **2. Map STL of intraoral scan with CBCT**  **3. Map STL of virtual crown with CBCT**  **4. Draw panoramic curve**  **5. Export STL of surgical stent**  **6. Check implant position**  **7. Draw surgical guide** | 1. 1,2,3,4,5,6,7 2. 2,3,4,1,6,7,5 3. 4,3,6,7,1,2,5 4. 2,6,7,4,3,5,1 |
|  | **Key answers- 1)-a, 2)-c, 3)-a, 4)-b, 5)-c, 6)-a, 7)-a, 8)-b**  **9)-d, 10)-b, 11)-a, 12)-b, 13)-a, 14)-a, 15)-b** |  |
